# The Challenge of Using an Antigen Test as a Screening Tool for SARS-CoV-2 Infection in an Emergency Department: Experience of a Tertiary Care Hospital in Southern Italy

**DOI:** 10.1155/2021/3893733

**Published:** 2021-09-11

**Authors:** Daniela Loconsole, Francesca Centrone, Caterina Morcavallo, Silvia Campanella, Anna Sallustio, Daniele Casulli, Marisa Accogli, Maria Chironna

**Affiliations:** ^1^Department of Biomedical Sciences and Human Oncology-Hygiene Section, University of Bari, P.zza G. Cesare 11, 70124 Bari, Italy; ^2^Azienda Ospedaliero-Universitaria Consorziale Policlinico di Bari, P.zza G. Cesare 11, 70124 Bari, Italy

## Abstract

**Background:**

In emergency hospital settings, rapid diagnosis and isolation of SARS-CoV-2 patients are required. The aim of the study was to evaluate the performance of an antigen chemiluminescence enzymatic immunoassay (CLEIA) and compare it with that of Real-time Reverse transcription-Polymerase Chain Reaction (RT-qPCR), the gold standard assay, to assess its suitability as a rapid diagnostic method for managing patients in the emergency department (ED).

**Methods:**

Consecutive patients with no previous history of SARS-CoV-2 infection attending the ED of the Policlinico Hospital of Bari between 23rd October and 4th November 2020 were enrolled. Clinical and demographic data were collected for all patients. Nasopharyngeal swabs collected on admission were subjected both to molecular (RT-qPCR) and antigen (CLEIA) tests for SARS-CoV-2. The performance of the CLEIA antigen test was analyzed using R Studio software and Microsoft Excel. Receiver operating characteristics were also performed.

**Results:**

A total of 911 patients were enrolled, of whom 469 (51.5%) were male. Of the whole cohort, 23.7% tested positive for SARS-CoV-2 by RT-qPCR and 24.5% by CLEIA. The overall concordance rate was 96.8%. The sensitivity, specificity, positive predictive value, and negative predictive value of the antigen test were 94.9% (95% CI, 91.9–97.0), 97.4% (95% CI, 96.5–98.1), 91.9% (95% CI, 89.0–94.0), and 98.4% (95% CI, 97.4–99.1), respectively. The area under the curve (AUC) was 0.99. The kappa coefficient was 0.91. The overall positive and negative likelihood ratios were 37 (95% CI 23-58) and 0.05 (95% CI, 0.03–0.09), respectively.

**Conclusions:**

Data analysis demonstrated that the antigen test showed very good accuracy for discriminating SARS-CoV-2-infected patients from negative participants. The CLEIA is suitable for rapid clinical diagnosis of patients in hospital settings, particularly in EDs with a high prevalence of symptomatic patients and where a rapid turnaround time is critical. Timely and accurate testing for SARS-CoV-2 plays a crucial role in limiting the spread of the virus.

## 1. Introduction

COVID-19 has affected more than 115 million people worldwide, and over 2.5 million deaths have been registered since the beginning of the pandemic [1]. Italy has suffered one of the harshest and earliest COVID-19 epidemics and is now experiencing a third wave. Large-scale nonpharmaceutical interventions such as the closure of schools and national lockdowns were implemented to contain the spread of the virus [2, 3]. Accurate diagnosis, isolation of cases and close contacts, and appropriate care for all patients with COVID-19 are the cornerstones of the pandemic containment measures [4]. The high number of cases reported in Italy is putting increased pressure on hospital systems, resulting in delays to treatment and stressing the national healthcare service. Real-time Reverse transcription-Polymerase Chain Reaction (RT-qPCR) is the gold standard diagnostic method for detecting SARS-CoV-2 infection [5]. However, RT-qPCR requires a BSL-2 (Bio Safety Level 2) laboratory and specialized personnel, and the results take a few hours [6]. However, unlike viral cultures, molecular tests do not allow differentiation of infectious from noninfectious virus [7]. Different studies show that the cycle threshold (Ct) value can be considered to be an indirect index of viral load in different specimens [7–9]. In particular, the Ct value is inversely proportional to the viral RNA copy number in a positive sample and so can be considered a valuable proxy for infectious virus [10–12]. Moreover, recent studies show that a high viral load, a low Ct value, and the number of days postsymptom onset are closely related [11, 13]. Singanayagam et al. estimated that the probability of culturing the virus decreases to 8% in samples with a Ct > 35 and to 6% after 10 days from symptom onset [10].

Emergency departments (ED) have been overwhelmed; therefore, the need for less laborious rapid tests with good sensitivity and specificity would ensure rapid diagnosis and isolation of infected individuals [14]. Recently, new tests with rapid turnaround times, such as the antigen tests, have been recommended in some countries [6, 15]. These antigen tests detect a specific SARS-CoV-2 antigen in samples collected from the upper respiratory tract [16]. Qualitative tests based on chromatography assays, and quantitative tests based on the CLEIA (chemiluminescence enzyme immunoassay) method, are available. The first can be used easily as a point of care test in settings other than hospitals, such as schools, airports, and nursing homes, since it does not require laboratory equipment, it is easy to handle, and it provides results within 15–30 minutes. The antigen test authorized for screening in settings other than hospital has been described [16]. The CLEIA antigen assay needs laboratory equipment, and results are available within 50–60 minutes. The CLEIA method is also used for the detection of other infectious diseases (e.g., Treponema pallidum infection) and for the detection of markers of noninfectious diseases (e.g., neurodegeneration).

Antigen tests are less sensitive than RT-qPCR [16]; therefore, they cannot be used as diagnostic tests, particularly in settings such as hospitals [13, 14, 16]. However, several studies show that this kind of test has high sensitivity if performed within 7 days after symptom onset [7, 11, 13], or when used to test samples that show a low Ct value in RT-qPCR tests [13].

The aim of the study was to evaluate the performance of the antigen CLEIA and compare it with that of RT-qPCR in patients attending the ED of a tertiary care hospital in Italy. The goal was to verify the CLEIA antigen test as a diagnostic method appropriate for diagnosing COVID-19 patients attending emergency hospital settings.

## 2. Materials and Methods

### 2.1. Study Population

The study population comprised all consecutive patients admitted to the ED of a tertiary care hospital (Policlinico Hospital-Giovanni XXIII Pediatric Hospital, Bari, Italy) between 23rd October and 4th November 2020. Patients without a previous history of SARS-CoV-2 infection were enrolled. At the time of admission, all patients were subjected to a nasopharyngeal swab test using a universal transport medium (UTM) (FLOQSwabs™, Copan Italia, Brescia, Italy). Samples were processed at the Laboratory of Molecular Epidemiology and Public Health of the Hygiene Unit of the Policlinico Hospital of Bari, which is the Regional Reference Laboratory for surveillance and diagnosis of SARS-CoV-2 infections. All samples were subjected both to an antigen test for SARS-CoV-2 (CLEIA, Fujirebio, Inc., Tokyo, Japan) and a molecular test (RT-qPCR assay) within 1 h of collection. Symptoms, the number of days since symptom onset, demographic data, and the Ct value of the RT-qPCR test were recorded for all patients.

For case definition, the National Institutes of Health (NIH) clinical staging of COVID-19 disease was used. In particular, all patients with a positive RT-qPCR for SARS-CoV-2 were classified as follows: asymptomatic infection (patients showed no signs or symptoms of COVID-19); mild illness (patients showed mild symptoms such as fever, cough, sore throat, malaise, headache, muscle pain, nausea, vomiting, diarrhea, loss of taste and smell, but without shortness of breath, dyspnea, or abnormal chest imaging); moderate illness (patients showed evidence of lower respiratory disease during clinical assessment but did not require hospitalization); severe illness (patients with SpO_2_ 30 breaths/min or lung infiltrates > 50% on chest imaging, with clear signs and symptoms of respiratory disease severe enough to require hospitalization); critical illness, (patients showing respiratory failure, septic shock, and/or multiple organ dysfunction and requiring admission to the intensive care unit) [17]. For the purposes of the study, cases of severe and critical illness were combined into a group labeled “severe illness.” Patients presenting at the ED with an illness other than COVID-19 were defined as “asymptomatic.”

### 2.2. CLEIA Antigen Test of SARS-CoV-2

The Lumipulse G SARS-CoV-2 Ag test (Fujirebio, Europe, Ghent, Belgium) is a completely automated assay with a throughput of 120 samples per hour; this CLEIA-based test is capable of detecting and quantitatively measuring the presence of SARS-CoV-2 nucleocapsid protein in nasopharyngeal swabs. To perform the test, samples were centrifuged at 2000 × g for 10 minutes and the supernatant was used for the analysis with the Lumipulse SARS-CoV-2 Ag kit (Fujirebio), which was then read by the Lumipulse G1200 automated immunoassay analyzer (Fujirebio). Briefly, the supernatant was aspirated using a single pipette tip, dispensed into the anti-SARS-CoV-2 Ag monoclonal antibody-coated magnetic particle solution, and incubated for 10 minutes at 37°C. After the first wash step, an alkaline phosphatase-conjugated anti-SARS-CoV-2 Ag monoclonal antibody was added for 10 min at 37°C. Following another wash step, the substrate solution was added for 5 minutes at 37°C. The resulting reaction signals are proportional to the amount of SARS-CoV-2 Ag in the sample, allowing quantitative determination of the amount of SARS-CoV-2 Ag in the nasopharyngeal swabs. According to the manufacturer's instructions, the test is negative when the antigen level is <1.34 pg/mL and positive when it is >10 pg/mL. Values between 1.34 pg/mL and 10 pg/mL were considered to be a “gray zone.” For analysis purposes, patients with an antigen test result in the gray zone were considered positive.

### 2.3. Molecular Identification of SARS-CoV-2

RNA was extracted using the MagMAX Viral/Pathogen Nucleic Acid Isolation kit (Thermo Fisher Scientific, Waltham, MA, USA) and the KingFisher Duo Prime System (Thermo Fisher Scientific). The molecular test was performed using the TaqPath RT-qPCR COVID-19 Assay, a three-target commercial multiplex RT-qPCR assay that identifies the N, ORF1ab, and S genes (Thermo Fisher Scientific). Results were interpreted according to the manufacturer's instructions. Samples with cycle threshold (Ct) values > 40 were considered negative. For each sample, the Ct values were recorded for all three genes and the average Ct value for each sample was used for analysis.

### 2.4. Data Analysis

The criteria used to assess the performance of the CLEIA antigen test were sensitivity, specificity, negative predictive value (NPV), positive predictive value (PPV), positive likelihood ratio (LR+), and negative likelihood ratio (LR-). Data analysis was performed using R Studio software (RStudio, Northern Ave, Boston, MA, USA) and Microsoft Excel (Microsoft Corp., Redmond, WA, USA). The results of the antigen test for each sample were also subjected to ROC (receiver operating characteristic) curve analysis. The AUC (area under the curve) was calculated based on a classification of the discriminating capacity of a test proposed by Swets, which is based on largely subjective criteria [18]. Results were compared with those from the RT-qPCR; therefore, samples classed as positive and negative by the molecular test were taken to be true positive and true negative samples. A *t*-test was used to compare differences between the average of Ct values in the CLEIA+ and CLEIA- group and to compare the average Ct values and antigen test values between asymptomatic and symptomatic patients. Pearson's correlation analysis was performed to assess the correlation between Ct values and antigen test values (log_10_ pg/mL). A *p* value ≤ 0.05 was considered statistically significant. The kappa coefficient was calculated to evaluate concordance between the two tests [19, 20].

### 2.5. Ethical Statement

Ethical approval was not required because the activities described were conducted as part of routine diagnostic tests. All procedures were carried out in accordance with the Declaration of Helsinki, as revised in 2013, for research involving human subjects. The data were deidentified; therefore, the need for informed consent was waived.

## 3. Results

A total of 911 patients were enrolled, of whom 469 (51.5%) were male. The characteristics and the distribution of patients are shown in Table 1. The clinical stage of all patients was known (Table 1); however, in the case of 46 patients, the number of days postsymptom onset was unknown. The average time between illness onset and sample collection was 4.5 days (Table 1).

In the overall study population, 216/911 patients (23.7%) tested positive for SARS-CoV-2 by RT-qPCR (RT-qPCR+), with an average Ct value of 22.9 (range, 11–36). Of all patients, 223/911 (24.5%) tested positive in the SARS-CoV-2 antigen test, with a mean value of 1,788.8 pg/mL (range, 1.34–5,000 pg/mL). The comparison between the average Ct values and the average antigen test values according to symptoms status is shown in Table 2.

All subjects with a positive antigen test (CLEIA+) were also positive in the RT-qPCR. Of those with a negative antigen test (CLEIA-), 11/688 (1.6%) were positive in the RT-qPCR, with an average Ct value of 32.1 (range, 26–35). Of these, four samples were collected from asymptomatic patients and seven from symptomatic patients, one of whom was clinically severe and whose sample was collected 3 days postsymptom onset. For 8/11 samples, the average Ct value was ≥30. For one of the 11 samples, the average Ct value was 25. This sample was collected 1 day from symptom onset, and the patient showed mild illness. The data regarding the antigen test and molecular tests for SARS-CoV-2 are shown in Table 3.

The RT-PCR-positive samples tested using the antigen test showed a median antigen level of 492.1 pg/mL (IQR, 28.9–5,000 pg/mL). The PCR-negative samples showed a median antigen level of 0.08 pg/mL (IQR, 0.04–0.14).

**Figure 1:**
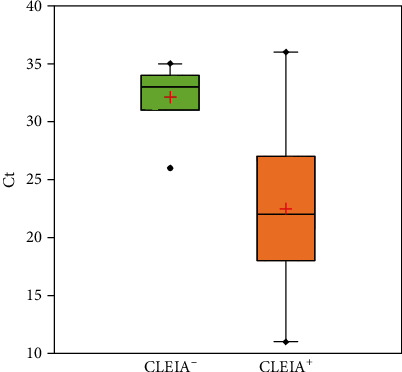
Box plot of the antigen test results versus the RT-qPCR Ct values of subjects with SARS-CoV-2 infection. Ct: cycle threshold.

**Figure 2:**
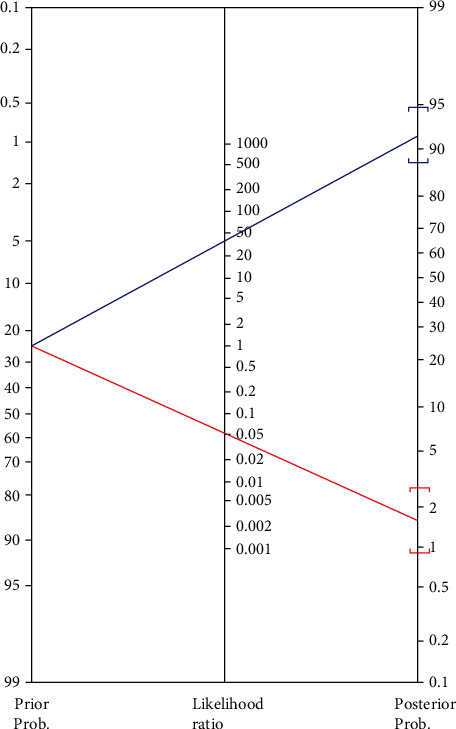
Nomogram of the positive (blue) and negative (red) likelihood ratio, and prior and posterior probabilities. Positive test: positive likelihood ratio: 37; 95% confidence interval: [23–58]; posterior probability (odds): 92% (11.5); 95% confidence interval: [88–95%]; (~1 in 1.1 with a positive test was sick). Negative test: negative likelihood ratio: 0.05; 95% confidence interval: [0.03–0.09]; posterior probability (odds): 2% (0.0); 95% confidence interval: [1–3%]; (~1 in 1.0 with a negative test was well).

Ct values were available for 192/216 patients with a positive RT-PCR result. Samples that were negative in the antigen test showed significantly higher values than the CLEIA+ specimens (*p* value < 0.00001) (Figure 1). Pearson's correlation analysis identified a strong negative correlation (*r* = −0.8579) between Ct and antigen test values (*p* < 0.00001). The coefficient of determination (*R*^2^) value was 0.736. The antigen test had an overall sensitivity of 94.9% (95% confidence interval (CI) 91.9–97.0), an overall specificity of 97.4% (95% CI, 96.5–98.1), and an overall concordance of 96.8%. Sensitivity, specificity, PPV, NPV, and test concordance, according to the presence of symptoms and time of symptom onset (≤7 days or >7 days), are reported in Table 4. The kappa coefficient was 0.91. The overall LR+ ratio was 37 (95% CI, 23–58), and the overall LR- ratio was 0.05 (95% CI, 0.03–0.09) (Figure 2).

**Figure 3:**
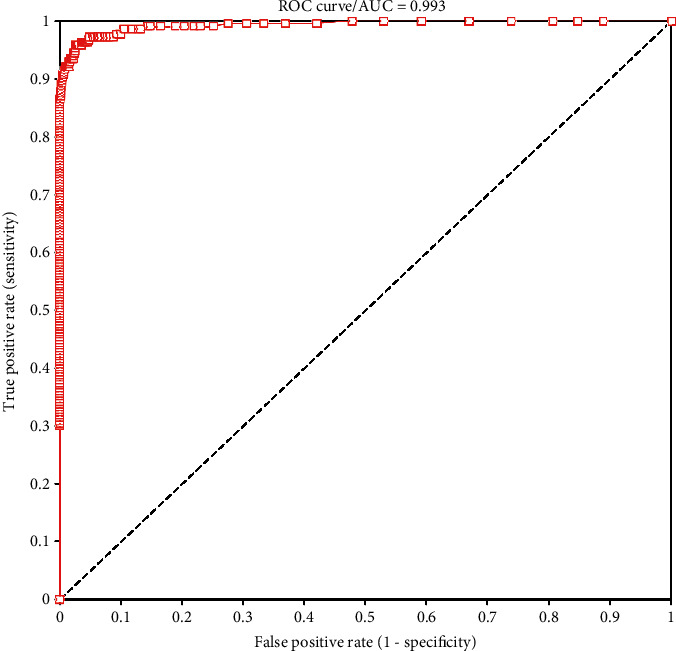
ROC curve for the chemiluminescence antigen test for SARS-CoV-2 antigen. ROC: receiver operating characteristic curve; AUC: area under the curve.

ROC curve analysis indicated that the optimal cut-off value for the Lumipulse assay for discriminating between positive and negative samples for SARS-CoV-2 infection was 1.25 pg/mL (Figure 3). The AUC value was 0.99.

## 4. Discussion

Timely and accurate testing for SARS-CoV-2 is crucial if we are to limit the spread of the virus. RT-qPCR remains the gold standard for diagnosis, but it is laborious and time-consuming. However, previous use of antigen tests to assess influenza viruses and syncytial respiratory virus infections shows us that this kind of test can be useful for detection of SARS-CoV-2. These tests are easy to handle, are inexpensive, and provide results in a short time [13]; however, their reliability in different settings needs to be ensured. In fact, the setting in which antigen tests are used largely determines their clinical performance [16]. Here, we evaluated the performance of a CLEIA antigen assay as a diagnostic tool to manage patients attending an ED. Since 60% of subjects presented at the ED had symptoms other than those typical of COVID-19 disease, it is crucial to identify correctly those that are true positives and put in place adequate prevention and control measures that prevent outbreaks and transmission in a hospital setting [16].

Both the antigen and RT-qPCR tests perform best when the subject has a high viral load [16, 21]. The European Center for Disease Control and Prevention (ECDC) recommends a minimum sensitivity for rapid antigen tests of 90% [13]. Based on the results of our study, the CLEIA antigen test showed a sensitivity of 95%, which is higher than that stated by the manufacturer (92%, based on 325 specimens). In our study, the test showed 92% sensitivity and 80% PPV among asymptomatic patients. Although these values are lower than those reported for symptomatic patients, the data are twofold higher than those reported by Pray et al. (41% sensitivity and 33% PPV among asymptomatic patients) [21]. However, the sensitivity of the antigen test was lower than that reported for RT-qPCR [16].

Our false positive rate was low; 92% of the CLEIA+ samples were confirmed by RT-qPCR. This is in accordance with data previously reported, since the specificity of the antigen tests is as high as most RT-qPCR tests [16].

We found that less than 2% of the results were false negatives when tested by RT-qPCR. These samples showed an average Ct value > 30. These results are attributed to the lower viral load in samples collected from these patients. The Ct values recorded in false negatives were significantly higher than those recorded for samples that tested positive in both the CLEIA and RT-qPCR tests. The concordance rate between the CLEIA and RT-qPCR assays was >96% for all samples, and 92% for samples obtained more than 7 days after symptom onset. In addition, the kappa coefficient revealed almost perfect agreement between the two tests. The optimal correlation between the antigen test and RT-qPCR was found at the early phase of infection. Symptomatic patients with a negative antigen test should be retested by RT-qPCR to limit the impact of false negatives (even though the concordance rate was high).

The data show that for patients tested at more than 7 days postsymptom onset, the specificity and the NPV were lower than those for patients tested within 7 days of symptom onset. This is in accordance with data reported by Hirotsu et al. [22]. Antigen tests are capable of detecting SARS-CoV-2 in samples harboring >100 RNA copies [22]; therefore, they are best used at the onset of infection, when the viral load is higher with higher probability of human-to-human transmission [22–24].

The positive and negative LR values may provide strong evidence that allows the ruling in or out of a diagnosis of SARS-CoV-2 infection (LR + >10 and LR − <0.1) [25]. For our population, the LR+ was 37, suggesting a very high probability that a patient with SARS-CoV-2 infection would test positive in the antigen test. Also, a study by Gili et al. reported that the LR+ was >10 [26].

ROC curve analysis demonstrated that the accuracy of the antigen test was almost perfect (AUC: 0.99) with respect to discriminating SARS-CoV-2-infected patients from negative subjects. Our findings are almost in line with the AUC value reported by Aoki et al. [27], and higher than that calculated by Hirotsu et al. [22] and Kobayashi et al. [28]. The optimal cut-off for positive samples was 1.25 pg/mL, lower than the 10.0 pg/mL reported by the manufacturer. Our data are almost the same as those reported by Gili et al. [26]. Using a cut-off value of 1.25 pg/mL instead of 1.34 pg/mL would have meant that two more samples that were positive in the RT-qPCR were also positive in the antigen test.

Antigen tests are increasingly being used for mass screening. Recently, novel SARS-CoV-2 virus variants of concern (VOCs), which are characterized by different mutations of the spike protein, have been identified [29]. In Italy, VOC 202012/01–B.1.1.7 lineage is circulating widely [30]. In the Apulia region, VOC 202012/01 was first detected at the end of December 2020 [31]; the estimated prevalence in March 2021 reached 93% [32]. The circulation of such a variant seems not to affect detection of SARS-CoV-2 by the antigen test, since these kinds of tests detect the N protein [33]. In the Apulia region, where the VOC 202012/01 has almost completely replaced wild-type SARS-CoV-2, the antigen assays continue to be used successfully to detect SARS-CoV-2 in settings other than hospitals. Moreover, prior COVID-19 vaccination will not affect the results of SARS-CoV-2 viral tests [34].

The automated Lumipulse assay showed high sensitivity. The results of our study suggest a possible use of this assay in hospital settings, in particular in the EDs. In fact, in settings with high prevalence of symptomatic patients (e.g., EDs), a positive antigen test would confirm an infection, while a negative result would lead to a RT-qPCR test for COVID-19 if the symptoms were consistent with disease. The high NPV suggests that asymptomatic patients attending the ED for a disease other than COVID-19 should be considered negative for SARS-CoV-2. This is crucial for the management of patients with COVID-19 who attend a hospital with no-COVID-19 wards.

Several factors may affect the accuracy of our study results. First, the high number of patients attending the ED means that an accurate epidemiological investigation to identify a possible exposure to a confirmed case of COVID-19 was not possible. Information about recent travel or strict contact with positive people was not available. Moreover, viral culture was not performed. However, the strength of the present study is that, compared with other studies, the study population was large and comprised of hospitalized patients without a prior diagnosis of SARS-CoV-2 infection. Therefore, we think that our results may be considered reliable.

## 5. Conclusions

Antigen tests could be a useful diagnostic tool in settings requiring a rapid test turnaround time. Such tests are low cost, are scalable, and provide results quickly; they also identify those who are likely to be harboring infectious virus. The accuracy of the CLEIA antigen assay means that it is suitable for clinical management of patients in hospital settings. In fact, even if false-negative results occurred, the reported Ct values were high, suggesting a very low probability of transmission of SARS-CoV-2 infection [10–12].

Optimizing the use of the CLEIA antigen assay could relieve the pressure on sanitary facilities due to the decrease in the number of samples that need to be confirmed by RT-qPCR. Thus, laboratories could perform a greater number of tests on samples from nonhospital settings.

## Figures and Tables

**Table 1:** Demographic and clinical characteristics of the SARS-CoV-2 positive patients.

*Total patients enrolled (N*)	911	%
Male	469	51.5%
Female	442	48.5%
Median age (IQR) (years)	52 (35–68)	
*COVID-19 symptoms and severity*		
Asymptomatic	550	60.4%
Symptomatic	361	39.6%
Mild	186	51.5%
Moderate	138	38.2%
Severe	37	10.3%
*Symptom onset (days)*		
Mean (range) (years)	4.5 (0–25)	

IQR: interquartile range.

**Table 2:** Comparison of the average Ct (cycle threshold) values and the average antigen test values (pg/mL) according to the symptom status.

	Asymptomatic	Symptomatic	*p* value
Average Ct values	22.35	23.09	0.442
Average CLEIA values (pg/mL)	1,642.94	1,837.69	0.563

**Table 3:** Comparison of the results from the CLEIA antigen test and the RT-qPCR test for SARS-CoV-2.

	RT-qPCR+ *N* (%)	RT-qPCR- *N* (%)	Total *N* (%)
CLEIA + (≥1.34 pg/mL)	205 (91.9%)	18 (8.1%)	223 (24.5%)
CLEIA - (<1.34 pg/mL)	11 (1.6%)	677 (98.4%)	688 (75.5%)
Total	216 (23.7%)	695 (76.3%)	911

RT-qPCR: Real-time Reverse transcription-Polymerase Chain Reaction; CLEIA: chemiluminescence enzyme immunoassay.

**Table 4:** Sensitivity, specificity, positive predictive value, negative predictive value, and concordance (%) of the CLEIA antigen test according to clinical picture and number of days postsymptom onset.

	Sensitivity (95% CI)	Specificity (95% CI)	PPV (95% CI)	NPV (95% CI)	Concordance (*N*)
All patients enrolled	94.9 (91.9–97.0)	97.4 (96.5–98.1)	91.9 (89.0–94.0)	98.4 (97.4–99.1)	96.8 (882/911)
Asymptomatic	91.8 (81.9–97.2)	97.8 (96.8–98.3)	80.4 (71.7–85.0)	99.2 (98.2–99.7)	97.3 (535/550)
Symptomatic	95.8 (92.7–97.7)	96.4 (93.7–98.0)	95.8 (92.7–97.7)	96.4 (93.7–98.0)	96.1 (347/361)
≤7 days	97.3 (93.4–99.1)	97.1 (94.1–98.6)	96.4 (92.6–98.2)	97.8 (94.8–99.3)	97.2 (242/249)
>7 days	93.6 (86.2–97.0)	89.5 (71.2–97.8)	95.7 (88.1–99.1)	85.0 (67.7–93.0)	92.4 (61/66)

Sensitivity: (CLEIA+ RT-qPCR+)/RT-qPCR+; specificity: (CLEIA- RT-qPCR-)/RT-qPCR-; PPV (positive predictive value): (CLEIA+ RT-qPCR+)/CLEIA+; NPV (negative predictive value): (CLEIA- RT-qPCR-)/CLEIA-; concordance: (CLEIA+ RT-qPCR+) + (CLEIA- RT-qPCR-)/(CLEIA+ RT-qPCR+) + (CLEIA- RT-qPCR-) + (CLEIA+ RT-qPCR-) + (CLEIA- RT-qPCR+).

## Data Availability

Data are available on request from the corresponding author.
